# A transcriptomic evaluation of the mechanism of programmed cell death of the replaceable bud in Chinese chestnut

**DOI:** 10.1515/biol-2022-0635

**Published:** 2023-07-07

**Authors:** Yan Guo, Shuhang Zhang, Ying Li, Xinfang Zhang, Huan Liu, Shiyuan Liu, Jing Liu, Guangpeng Wang

**Affiliations:** Chestnut Department, Changli Research Institute of Fruit Trees, Hebei Academy of Agricultural and Forestry Sciences, Changli, Hebei, 066600, China

**Keywords:** Chinese chestnut, replaceable bud, programmed cell death, transcriptomics

## Abstract

Previous studies suggest that the senescence and death of the replaceable bud of the Chinese chestnut cultivar (cv.) “Tima Zhenzhu” involves programmed cell death (PCD). However, the molecular network regulating replaceable bud PCD is poorly characterized. Here, we performed transcriptomic profiling on the chestnut cv. “Tima Zhenzhu” replaceable bud before (S20), during (S25), and after (S30) PCD to unravel the molecular mechanism underlying the PCD process. A total of 5,779, 9,867, and 2,674 differentially expressed genes (DEGs) were discovered upon comparison of S20 vs S25, S20 vs S30, and S25 vs S30, respectively. Approximately 6,137 DEGs common to at least two comparisons were selected for gene ontology (GO) and Kyoto Encyclopedia of Genes and Genomes (KEGG) enrichment analyses to interrogate the main corresponding biological functions and pathways. GO analysis showed that these common DEGs could be divided into three functional categories, including 15 cellular components, 14 molecular functions, and 19 biological processes. KEGG analysis found that “plant hormone signal transduction” included 93 DEGs. Overall, 441 DEGs were identified as related to the process of PCD. Most of these were found to be genes associated with ethylene signaling, as well as the initiation and execution of various PCD processes.

## 1 Introduction

Chinese chestnut (*Castanea mollissima* BL.) is an important nut-producing tree grown in temperate regions worldwide [1]. However, chestnut trees suffer reduced productivity over time due to the characteristics of branch growth. In chestnut trees, the apical bud of each existing fruiting branch will develop into a new fruiting branch in the following year, resulting in the majority of fruit development taking place along the peripheral crown. A notable exception, the spontaneous mutant chestnut cultivar (cv.) “Tima Zhenzhu,” was first described in 1979. In “Tima Zhenzhu” trees, the apical bud (hereafter “replaceable bud”) senesces and dies, resulting in a compact crown [2]. Research indicates that the death of the replaceable bud involves programmed cell death (PCD), as evidenced by the presence of certain markers of PCD, including chromatin condensation, nuclear degradation, DNA laddering, tonoplast invagination, vacuolar rupture, and autophagy, among others [1].

PCD, an orderly process of cellular suicide mediated by intracellular death programs [3], is crucial for normal tissue development and stress response [4]. In plants, developmental PCD regulated by internal factors occurs concomitantly during reproductive and vegetative development including cell death of the replaceable bud, root cap cells, nucellar tissue, and tapetum, as well as trichome formation, sex determination, endosperm, embryonic suspensor, xylogenesis, organ senescence, and aerenchyma formation [1,5,6,7]. Across diverse PCD cases, a common regulatory network coordinated among PCD preparation, initiation, effectors, and degradation has been documented in the regulatory mechanism of plant PCD [7]. Cellular preparation for PCD is coordinated primarily by transcriptional regulation of hormone signaling, most commonly of the ethylene signaling pathway [8,9,10,11]. Transcription factors (TFs) link phytohormone signaling to PCD regulation [12]. Downstream of hormone signaling, PCD can be triggered in specific cell types by diverse cellular events such as changes in intracellular Ca^2+^ concentration, buildup of reactive nitrogen species and reactive oxygen species (ROS), activation of protein kinases, acidification of the cytoplasm, and modification of the cytoskeleton [13]. In addition, a plethora of hydrolytic enzymes, including nucleases and proteases, have been suggested as putative PCD executors [5,6,14].

In the present study, we performed transcriptomic profiling of the replaceable bud of Chinese chestnut cv. “Tima Zhenzhu” before (S20), during (S25), and after (S30) PCD. This study aimed to (i) investigate the expression patterns of the differentially expressed genes (DEGs) as well as their functions and metabolism pathways, and (ii) elucidate the regulatory and signaling pathways related to replaceable bud PCD in chestnut cv. “Tima Zhenzhu.” The results of this work will not only bolster our understanding of the molecular mechanism of replaceable bud PCD but may also provide a relevant resource for the future genetic improvement of chestnuts.

## 2 Materials and methods

### 2.1 Plant materials

**Figure 1:**
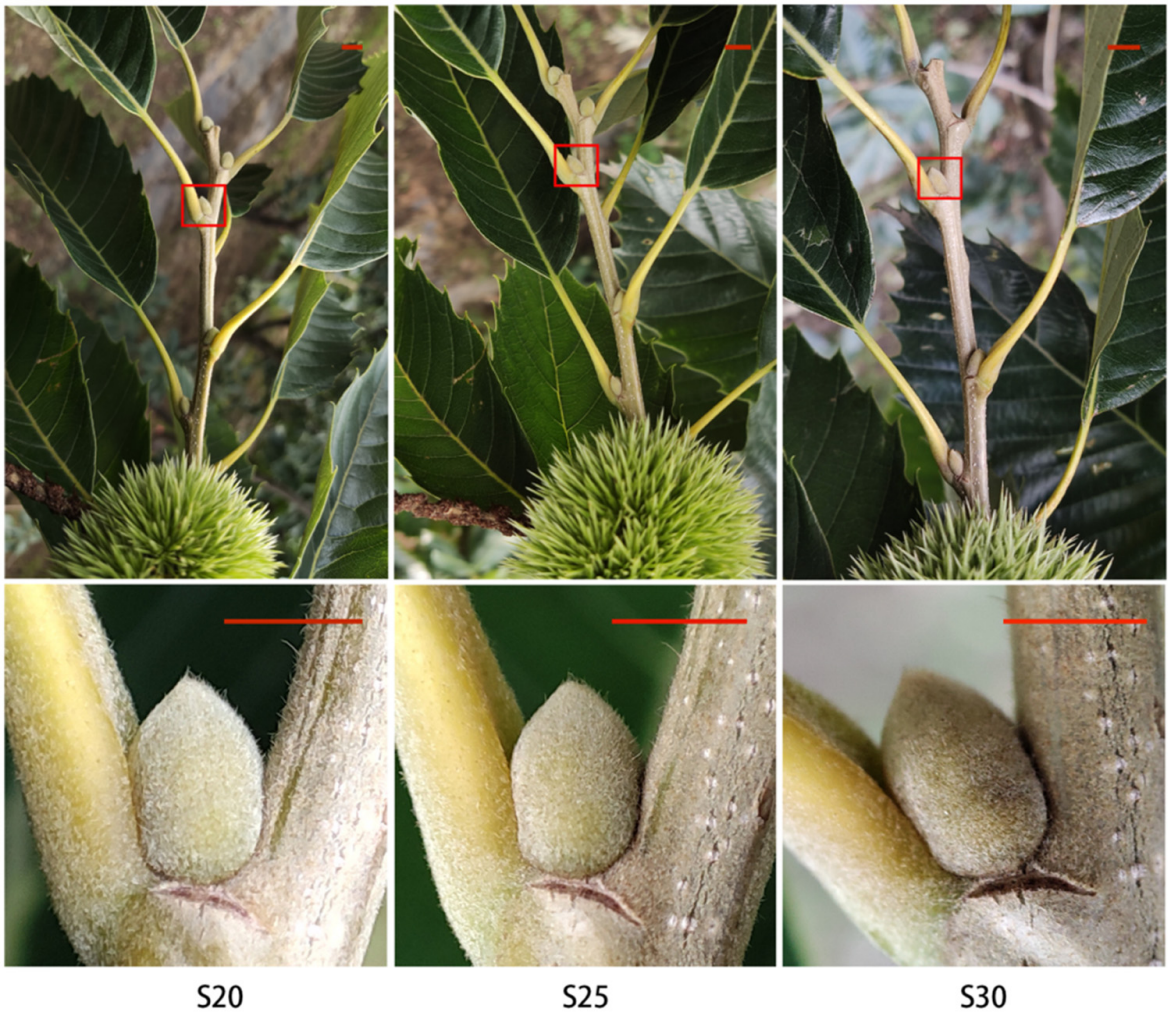
The process of replaceable bud senescence in Chinese chestnut cv. “Tima Zhenzhu.” S20: 20 days after flowering, before PCD, green buds. S25: 25 days after flowering, during PCD, yellow-green buds but no abscission layer formed. S30: 30 days after flowering, after PCD, brown-yellow buds with partial abscission layer formation. Bars = 6 mm.

Chestnut cv. “Tima Zhenzhu” (*C. mollissima BL.*) trees were kept at the Changli Institute of Pomology, Academy of Agricultural and Forestry Sciences, Hebei Province, China (118°51′ E, 39°53′ N). Tree age 12 years, row spacing 4 m × 4 m. A total of 15 trees with normal growth and no pests and diseases were selected. Based on previous research results, replaceable buds of the first three nodes of the top of the bearing branches with the same thickness and length were collected at 20 (S20), 25 (S25), and 30 (S30) days after flowering, corresponding to the developmental stage before, during, and after PCD (Wang et al. 2012). At S20, the buds are green and display a normal phenotype; at S25, the buds are yellow-green but no abscission layer formed; at S30, the buds are brown-yellow and display a partial abscission layer (Figure 1). Three biological replicates were collected at each phase, and each biological replicate contains 60 buds from 5 trees (a total of 15 trees).

### 2.2 Methods

#### 2.2.1 Transcriptomic sequencing and library construction

RNA-seq was carried out with 1 μg of RNA per sample. A NanoDrop 2000 spectrophotometer (Thermo Fisher Scientific, MA, USA) was utilized to ascertain both the purity and concentration of the RNA. An RNA Nano 6000 Assay Kit and a 2100 Bioanalyzer (Agilent Technologies, CA, USA) were utilized to ascertain RNA integrity. Transcriptomic analysis was conducted by the Biomarker Technologies Company (Beijing, China). An NEBNext Ultra RNA Library Prep Kit for Illumina (New England Biolabs, MA, USA) was utilized to generate sequencing libraries. An AMPure XP system (Beckman Coulter, CA, USA) was utilized to perform the purification of library fragments. An Illumina NovaSeq 6000 platform (Illumina, CA, USA) was utilized to perform library sequencing.

#### 2.2.2 Identification and functional annotation of DEGs

Raw sequence data were filtered by removing adapter sequences and low-quality reads. HISAT2 software [15] was utilized to map clean reads to the *C. mollissima* reference genome [16]. StringTie software [17] was utilized to quantify gene expression as fragments per kilobase of exon per million mapped reads (FPKM) values. Genes were considered as “expressed” if their FPKM was >0. DESeq2 software [18] was utilized to discover DEGs between the S20, S25, and S30 time points, with a threshold false discovery rate (FDR) set at <0.01 and |log2FC| > 1. Finally, the Database for Annotation, Visualization, and Integrated Discovery (DAVID) was utilized for both analysis of Kyoto Encyclopedia of Genes and Genomes (KEGG) pathway enrichment and annotation of gene ontology (GO), as implemented in BMK Cloud provided by Biomarker Technologies Company (Beijing, China).

#### 2.2.3 Protein–protein interaction network construction

Protein–protein interactions were queried using the STRING database (https://string-db.org/cgi). The proteins encoded by DEGs putatively involved in replaceable bud PCD were networked using their respective tobacco homologs. The Cytoscape software [19] was utilized to visualize the resulting protein–protein interaction network.

#### 2.2.4 Quantitative real-time polymerase chain reaction (qRT-PCR) validation

An RNAqueous Total RNA Isolation Kit (Solebo Technology, China) was utilized to extract total RNA from the replaceable bud before (S20), during (S25), and after (S30) PCD. The HiScript II Q RT SuperMix for qPCR (Yisheng Biotechnology, China) was utilized to synthesize cDNA from the extracted RNA, with *Actin* serving as the internal reference gene. All qRT-PCR primers can be found in Table S7. A LightCycler 480II Real-Time PCR Detection System (Roche, Switzerland) was utilized to perform qRT-PCR. The reaction mixture contained 2 µL of the cDNA template, 10 μL of the ChamQ SYBR qPCR Master Mix, 6.8 μL of deionized water, 0.4 μL of ROX Reference Dye2, 0.4 µL of the forward primers, and 0.4 µL of the reverse primers. The amplification program was conducted as follows: 95°C for 5 min; 40 cycles of 95°C for 10 s, and 60°C for 30 s. To ensure data reproducibility, three independent experiments were conducted. The 2^−△△Ct^ method [13] was utilized to quantify the levels of relative gene expression.

## 3 Results

### 3.1 Transcriptomic analysis

**Figure 2:**
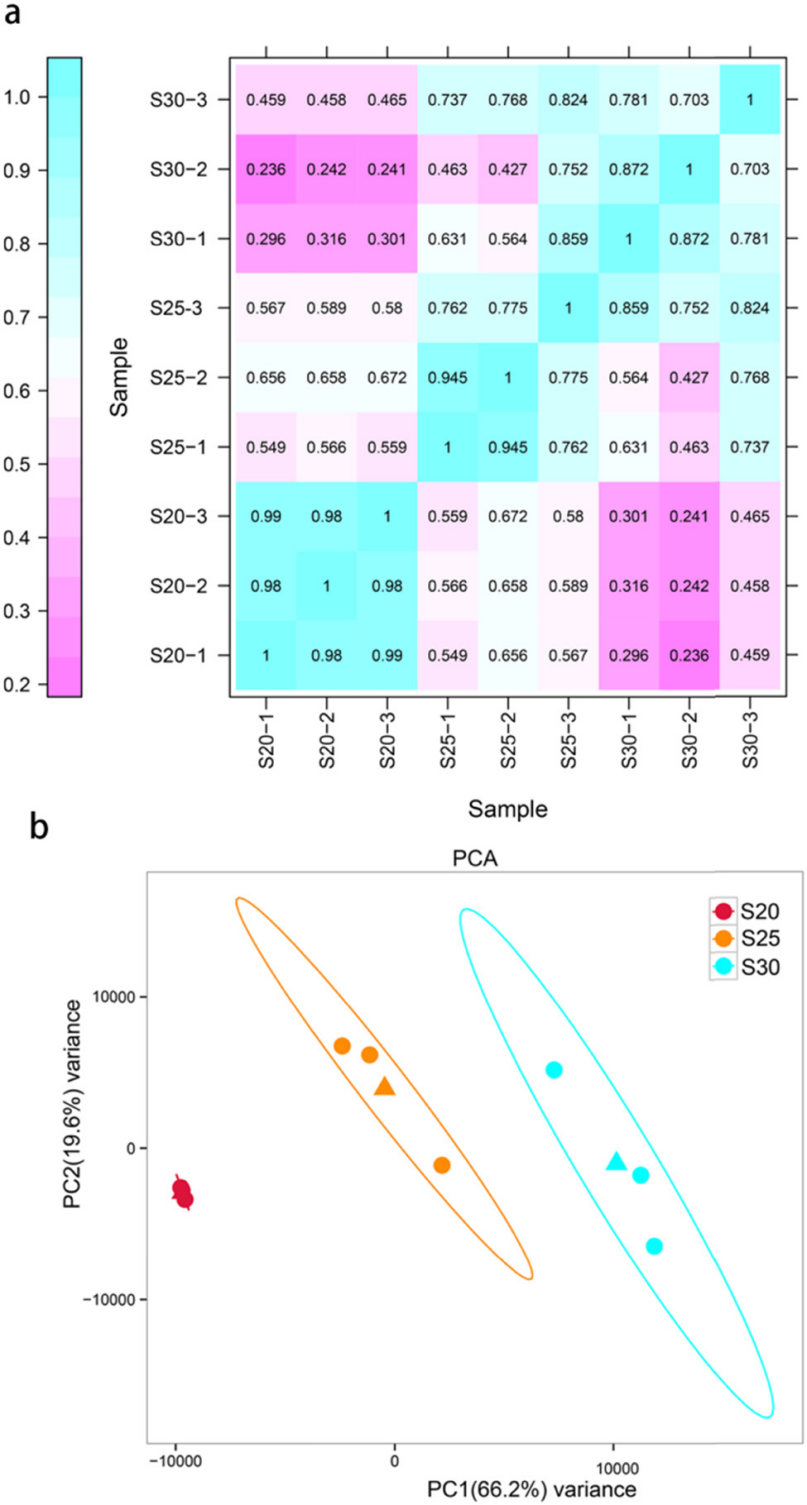
Assessment of the relationships among different replaceable bud samples of chestnut cv. “Tima Zhenzhu” using heat mapping (a) and PCA (b), with each sample (S20, S25, and S30) containing three biological replicates (−1, −2, and −3). The triangle represents the coordinates (center point) of the mean of the three biological replicates for each group of samples.

Samples of chestnut cv. “Tima Zhenzhu” replaceable bud tissue were used to prepare RNA libraries before (S20), during (S25), and after (S30) PCD. After quality control, clean reads were mapped to the *C. mollissima* reference genome, producing 86.94% (37,370,780), 90.25% (40,861,735), and 89.77% (40,896,455) mapped reads in the S20, S25, and S30 libraries, respectively (Table 1). Of these, uniquely mapped reads accounted for 82.87% (35,620,696) of the S20 library, 85.43% (38,678,320) of the S25 library, and 83.21% (37,872,455) of the S30 library. The rate of multiple mapped reads across the three libraries was less than 6.56%, and the percentage of Q30 bases was 93.24%, 94.29%, and 93.31% in the S20, S25, and S30 libraries, respectively (Table 1), indicating that our data were of sufficiently high quality. To assess the reliability of the constructed libraries, heat mapping and principal component analysis (PCA) were used to examine relationships among different samples and replicates (Figure 2). Overall, the read counts between both samples and replicates showed high correlations, with correlation coefficients (*r*) ranging between 0.98–0.99, 0.775–0.945, and 0.703–0.872 in S20, S25, and S30, respectively (Figure 2a). Significant differences were observed between samples (S20, S25, and S30), with a variance of 66.2% between samples and a variance of 19.6% within the same sample (Figure 2b).

**Table 1:** Survey of the RNA-seq results obtained from the replaceable bud of chestnut cv. “Tima Zhenzhu”

Sample	S20	S25	S30
Total reads	42,979,831	45,269,139	45,562,945
Total mapped reads	37,370,780 (86.94%)	40,861,735 (90.25%)	40,896,455 (89.77%)
Uniquely mapped reads	35,620,696 (82.87%)	38,678,320 (85.43%)	37,872,455 (83.21%)
Multiple mapped Reads	1,750,084 (4.07%)	2,183,415 (4.81%)	3,024,000 (6.56%)
GC content (%)	44.92	44.82	45.11
Percentage of Q30 base	93.24	94.29	93.31

### 3.2 Global transcriptional changes before, during, and after PCD in replaceable buds of chestnut cv. “Tima Zhenzhu”

**Figure 3:**
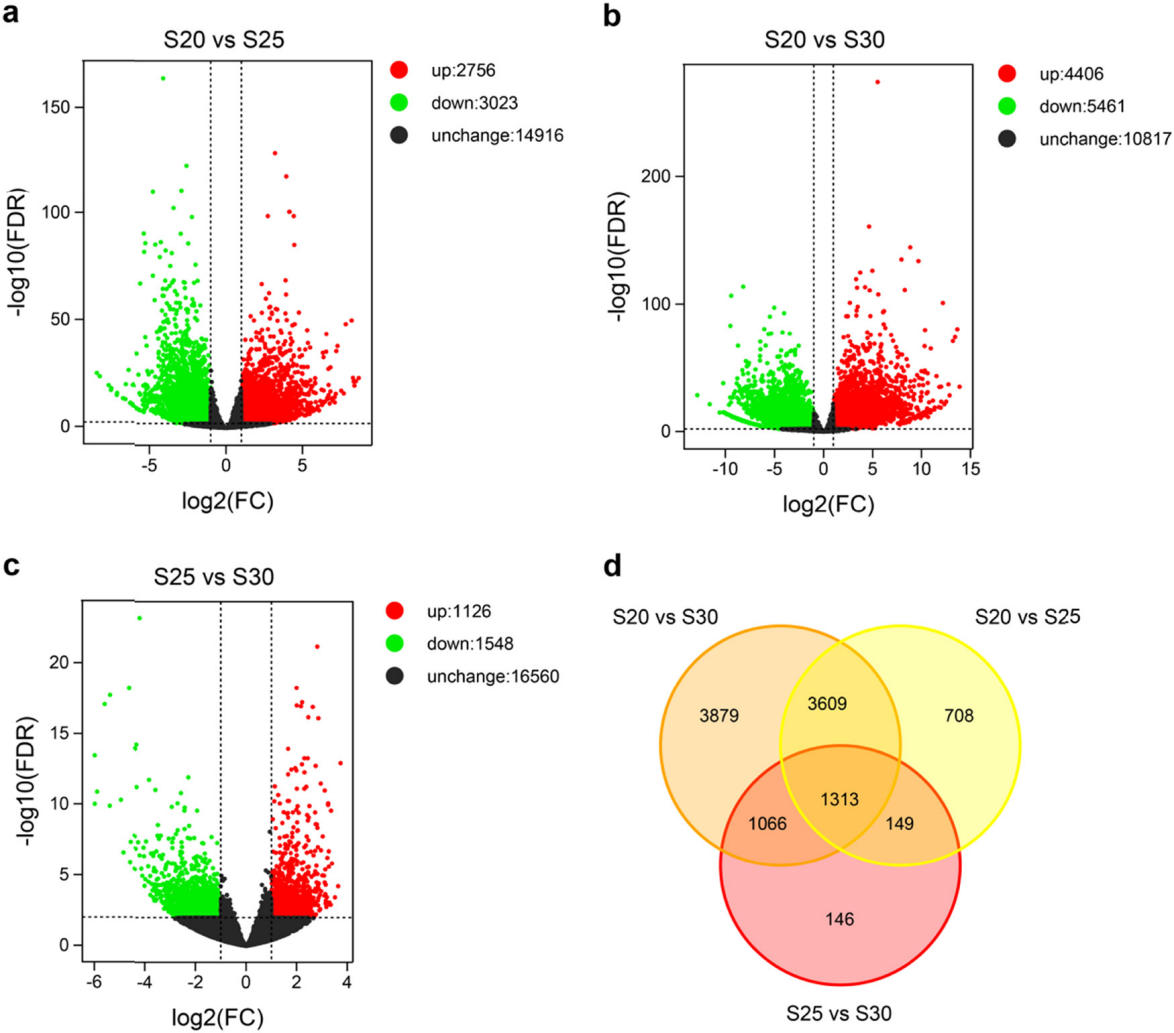
(a–c) Expression profiles of DEGs in three comparison groups. Green and red dots represent down- and upregulated DEGs with FDR ≤ 0.01 and |log_2_(FC)| > 1, respectively. Black dots indicate genes without expression changes. FC (fold change) was calculated based on the FPKM values in S20 vs S25, S20 vs S30, and S25 vs S30. (d) Venn diagram of DEGs in three comparison groups.

In order to identify DEGs, a stringent threshold of FDR < 0.01 and |log_2_FC| > 1 was used. In total, 5,779 DEGs were identified in S20 vs S25, 9,867 in S20 vs S30, and 2,674 in S25 vs S30 (Figure 3). The large number of DEGs (10,870 in total; Table S1) suggested that replaceable bud PCD may be a complex process regulated by an extensive population of genes. Moreover, we found a significantly greater number of DEGs in S20 vs S30, compared to the other two timepoint comparisons, indicating that gene expression in replaceable bud PCD is highly variable across time. Clear differences were found in global gene expression patterns between the three time points (Figure 3). In general, the amount of both down- and upregulated genes was large in all comparisons, although the amount of down-regulated genes tended to be slightly higher (Figure 3).

### 3.3 Functional enrichment analysis of the common DEGs

**Figure 4:**
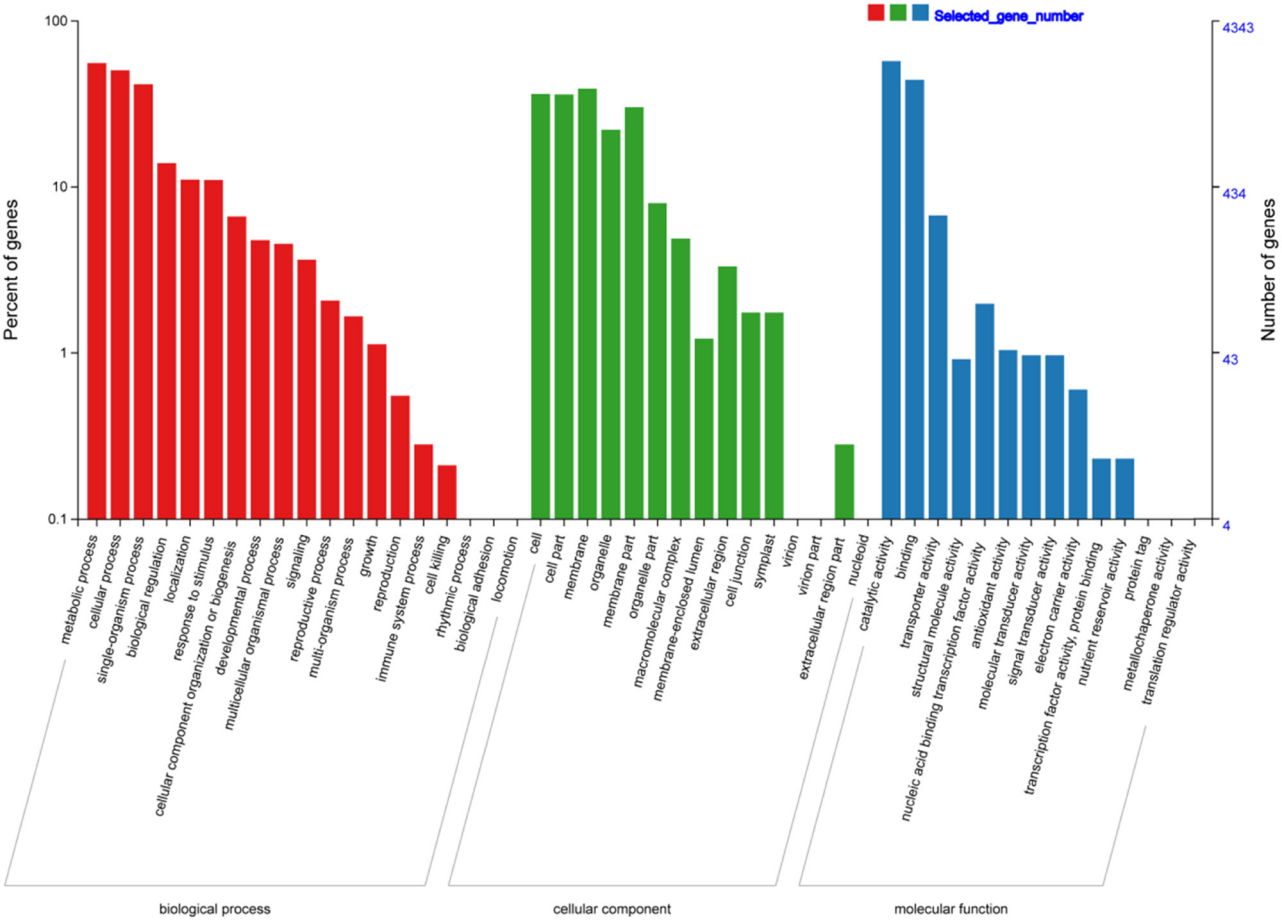
GO classification of the 6,137 common DEGs. GO terms were divided into three categories: molecular functions, BPs, and CCs.

The DEGs shared by at least two comparisons (“common DEGs”; 6,137 in total) were further evaluated to assess their core biological functions related to replaceable bud PCD (Figure 3d; Table S2). The cross-DEGs were annotated with 19 biological processes (BPs), 15 cellular components (CCs), and 14 molecular functions in GO categories (Figure 4), and significantly enriched (*P* ≤ 0.001) into 35 GO terms (Table S3). “Microtubule cytoskeleton organization” (GO:0000226), “oxidation–reduction process” (GO:0055114), “lignin catabolic process” (GO:0046274), “protein phosphorylation” (GO:0006468), “cell wall macromolecule catabolic process” (GO:0016998), “metabolic process” (GO:0008152), “microtubule-based movement” (GO:0007018), “carbohydrate metabolic process” (GO:0005975), and “regulation of transcription, DNA-templated” (GO:0006355) were dominant terms in BP; “integral component of membrane” (GO:0016021), “anchored component of plasma membrane” (GO:0046658), and “microtubule” (GO:0005874) were of the representative terms in CC. Among MF, a great number of DEGs were enriched in “transferase activity, transferring acyl groups other than amino-acyl groups” (GO:0016747), “microtubule motor activity” (GO:0003777), “polysaccharide binding” (GO:0030247), “protein serine/threonine kinase activity” (GO:0004674), and “hydrolase activity, hydrolyzing O-glycosyl compounds” (GO:0004553).

Several GO terms associated with either autophagy or PCD were identified (Table S3), including “negative regulation of programmed cell death” (GO:0043069), “singlet oxygen-mediated programmed cell death” (GO:0010343), “positive regulation of programmed cell death” (GO:0043068), “poly(A)-specific ribonuclease activity” (GO:0004535), “positive regulation of autophagy” (GO:0010508), “regulation of programmed cell death” (GO:0043067), “autophagy” (GO:0006914), “regulation of autophagy” (GO:0010506), “autophagy of mitochondrion” (GO:0000422), “autophagy of nucleus” (GO:0044804), “ribonuclease activity” (GO:0004540), and “3′-5′-exoribonuclease activity” (GO:0000175).

**Figure 5:**
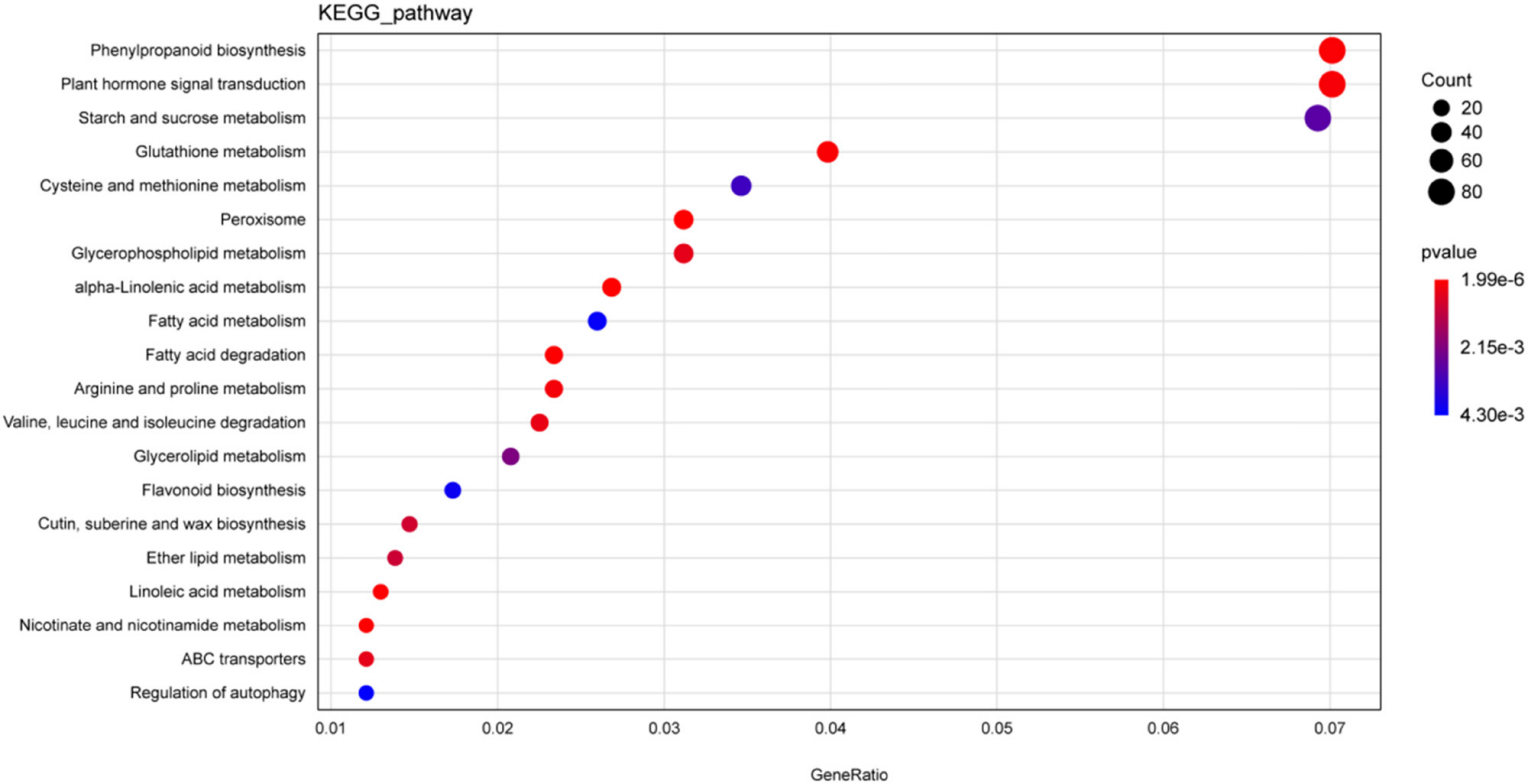
Top 20 enriched KEGG pathways of the 6,137 common DEGs. Coloring indicates lower (red) or higher (blue) significance (*P*-value). The dot size is proportional to the DEG number.

KEGG enrichment analysis was also conducted on the common DEGs in order to ascertain which biological pathways they may be related to (Table S4). Among the 125 enriched KEGG pathways identified, the most significant pathways (*P* ≤ 0.001) were “plant hormone signal transduction” (ko04075), “peroxisome” (ko04146), “linoleic acid metabolism” (ko00591), “alpha-linolenic acid metabolism” (ko00592), “nicotinate and nicotinamide metabolism” (ko00760), “phenylpropanoid biosynthesis” (ko00940), “glutathione metabolism” (ko00480), and “glycerophospholipid metabolism” (ko00564). In addition, the pathway “autophagy pathway” closely related to PCD was also identified ( although it did not reach a significant enrichment level) (Table S4 and Figure 5).

### 3.4 DEGs related to phytohormone signal transduction pathways

**Figure 6:**
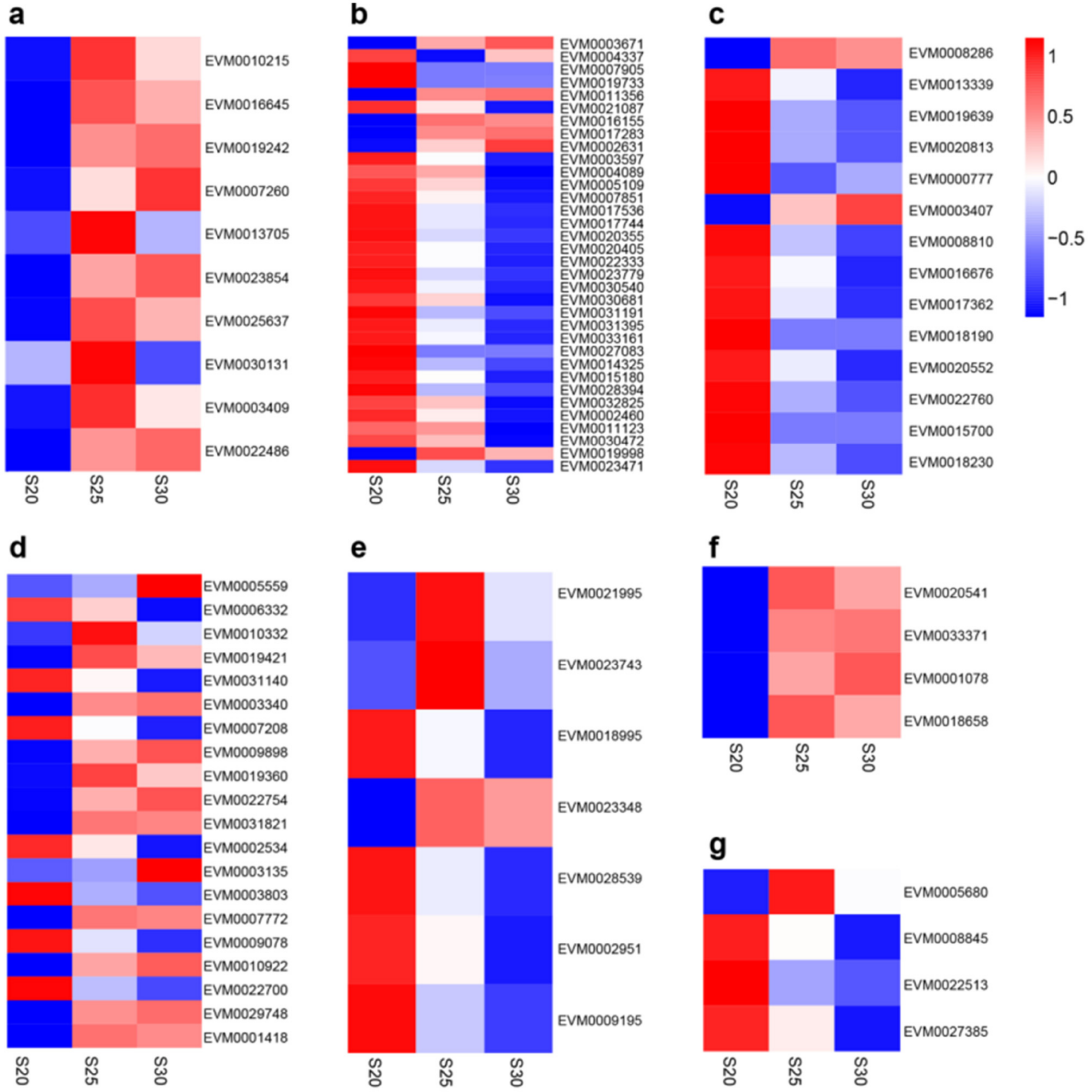
Heat maps showing phytohormone-related DEG expression patterns, including ethylene (a), auxin (b), cytokinin (c), ABA (d), GA (e), SA (f), and BR (g).

In total, the “plant hormone signal transduction” (ko04075) KEGG pathway was found to contain 93 DEGs (Table S5), the bulk of which were related to the ethylene, BR, SA, GA, ABA, cytokinin, or auxin signaling pathways (Figure 6). Within the ethylene pathway, the following DEGs were upregulated at S25 and S30, compared to S20: EVM0010215 (encoding mitogen-activated protein kinase like d5), EVM0016645 (encoding ethylene response sensor 1), EVM0019242 (encoding ethylene receptor 2-like), EVM0007260 (encoding EIN3-binding F-box protein), three DEGs encoding ethylene-insensitive protein 3 (EVM0013705, EVM0023854, and EVM0025637), and three DEGs encoding ethylene-responsive TFs (EVM0030131, EVM0003409, and EVM0022486). In the auxin pathway, the following DEGs were downregulated at S25 and S30, compared to S20: 15 DEGs encoding auxin-responsive proteins (IAA) (e.g., EVM0003597, EVM0004089, and EVM0005109), four DEGs encoding auxin response factors (EVM0015180, EVM0028394, EVM0032825, and EVM0002460), two DEGs encoding auxin-induced proteins (EVM0027083 and EVM0014325), and two DEGs encoding auxin transporter-like proteins (EVM0011123 and EVM0030472). Additionally, in the auxin pathway, EVM0019998, encoding an auxin influx transport protein, was upregulated at S25 and S30, compared to S20. Moreover, five up- and four downregulated DEGs encoded auxin-responsive protein SAUR genes (e.g., EVM0003671, EVM0004337, and EVM0007905). In the cytokinin pathway, one DEG (EVM0008286) encoding a histidine kinase and one DEG (EVM0003407) encoding two-component response regulators were upregulated, while three DEGs (EVM0013339, EVM0019639, and EVM0020813) encoding histidine kinases, eight DEGs (e.g., EVM0000777, EVM0008810, EVM0016676, and EVM0017362) encoding two-component response regulators, and two DEGs (EVM0015700 and EVM0018230) encoding histidine-containing phosphotransfer proteins were downregulated at S25 and S30, compared to S20. In the ABA pathway, 12 DEGs were upregulated, and 7 DEGs were downregulated at S25 and S30, compared to S20, among which four DEGs encoded ABA-insensitive 5-like proteins (two up- and two downregulated: EVM0006332, EVM0010332, EVM0019421, and EVM0031140), six DEGs encoded protein phosphatase 2 C (one down- and five upregulated; e.g., EVM0003340, EVM0007208, and EVM0009898), and nine DEGs encoded serine/threonine–protein kinases (four down- and five upregulated; e.g., EVM0002534, EVM0003135, and EVM0003803). In the GA pathway, two DEGs encoding gibberellin receptors (EVM0018995 and EVM0023348) and two DEGs encoding DELLA proteins (EVM0002951 and EVM0009195) were downregulated, whereas two DEGs encoding TF PIF3 (EVM0021995 and EVM0023743) were upregulated at S25 and S30, compared to S20. In the SA pathway, three DEGs encoding pathogenesis-related protein 1-like (EVM0020541, EVM0033371, and EVM0001078) and EVM0018658, encoding a regulatory protein, were consistently upregulated. In the BR pathway, several DEGs encoding brassinosteroid insensitive 1-associated receptor kinase 1 were up- (e.g., EVM0005680) or downregulated (e.g., EVM0008845, EVM0022513, and EVM0027385 for cyclin-D3).

### 3.5 DEGs related to replaceable bud PCD regulation in chestnut cv. “Tima Zhenzhu”

To identify DEGs associated with replaceable bud PCD in chestnut cv. “Tima zhenzhu,” the functional annotations of all DEGs were blasted against the NCBI non-redundant (NR) protein sequences database. A total of 441 DEGs were found to be related to PCD processes, including hormone signaling pathways, PCD initiation, and PCD execution (Table S6). In the present study, the ethylene signaling pathway was one of the significantly enriched KEGG pathways and involved ten DEGs described in Section 3.4. Moreover, a total of 16 DEGs related to ethylene biosynthesis exhibited significantly higher expression levels at S25 and S30 compared to S20, including 3 1-aminocyc lopropane-1-carboxylate synthases (EVM0001055, EVM0011816, and EVM0029965), 1 s-adenosylmethionine synthase (EVM0032964), and 12 1-aminocyclopropane-1-carboxylate oxidases (e.g., EVM0000849, EVM0006088, EVM0006661, and EVM0010524).

Both PCD initiation and execution require the temporal and spatial coordination of many precisely regulated processes, which work downstream of ethylene signaling [13]. We found that DEGs related to PCD initiation were primarily TFs, including 43 NAC TFs (e.g., EVM0010588, EVM0016926, and EVM0018368), 7 MADS-box TFs (e.g., EVM0019787, EVM0021142, and EVM0023741), 31 bHLH TFs (e.g., EVM0012487, EVM0013783, EVM0016064, and EVM0019464), and 49 MYB TFs (e.g., EVM0021418, EVM0024602, EVM0033473, and EVM0006679) (Table S6). Other functional components were also identified, including cytochrome c (EVM0024076), MAPK (EVM0012332, EVM0022934, EVM0010215, and EVM0030224), MAPKK (EVM0004329, EVM0000127, and EVM0025130), MAPKKK (e.g., EVM0015125, EVM0014496, and EVM0033128), and Ca^2+^ uniporters (EVM0018926 and EVM0024216). A total of 36 DEGs were associated with Ca^2+^ signaling (Table S6), including 4 Ca^2+^ channels (EVM0026184, EVM0007035, EVM0016547, and EVM0023315), 9 Ca^2+^ -dependent protein kinases (e.g., EVM0022389, EVM0025960, EVM0030576, and EVM0031549), 9 Ca^2+^ B-like proteins (e.g., EVM0000079, EVM0000956, EVM0004877, and EVM0010534), and 14 calmodulins (e.g., EVM0030734, EVM0033478, EVM0002917, and EVM0004863). DEGs related to PCD execution were primarily enriched in autophagy and proteases. Protease genes included 1 metacaspase (EVM0011448), 8 cysteine proteinases (e.g., EVM0007262, EVM0007929, EVM0020838, and EVM0025298), 2 vacuolar-processing enzymes (EVM0002134 and EVM0012232), 20 vacuolar protein sorting-associated proteins (e.g., EVM0012501, EVM0012829, EVM0013147, and EVM0015898), 9 endonucleases (EVM0006680, EVM0015983, EVM0021509, and EVM0022275), 19 aspartic proteases (e.g., EVM0005045, EVM0005145, EVM0009402, and EVM0012500), 5 endoglucanases (EVM0016472, EVM0017671, EVM0022249, EVM0032182, and EVM0003400), two senescence-associated proteins (EVM0009758 and EVM0031003), 17 pectinesterases (e.g., EVM0005897, EVM0021830, and EVM0032186), 13 xyloglucan endotransglucosylase/hydrolases (e.g., EVM0023103, EVM0028198, and EVM0031923), 13 lipoxygenases (e.g., EVM0032274, EVM0000380, EVM0000461, and EVM0002948), and 16 acid phosphatases (e.g., EVM0006765, EVM0014183, and EVM0022260). Autophagy-related DEGs included 18 autophagy-related proteins (e.g., EVM0032306, EVM0002553, EVM0003991, and EVM0004113), 7 protein-transport proteins (e.g., EVM0001633, EVM0007575, and EVM0011846), 1 syntaxin-related protein KNOLLE (EVM0031095), 2 vesicle transport proteins (EVM0014337 and EVM0016934), and one calreticulin (EVM0014858). Additionally, some DEGs involved in longevity regulation and stress response were identified, including 7 chaperonins (e.g., EVM0008263, EVM0008832, and EVM0009026), 6 BAG family molecular chaperone regulators (e.g., EVM0001410, EVM0009410, and EVM0024418), 2 catalases (EVM0024729 and EVM0009488), 3 Cu–Zn superoxide dismutases (EVM0015967, EVM0017860, and EVM0032156), 1 Mn superoxide dismutase (EVM0018305), and 37 heat-shock proteins (e.g., EVM0015018, EVM0015246, EVM0016538, and EVM0017835).

**Figure 7:**
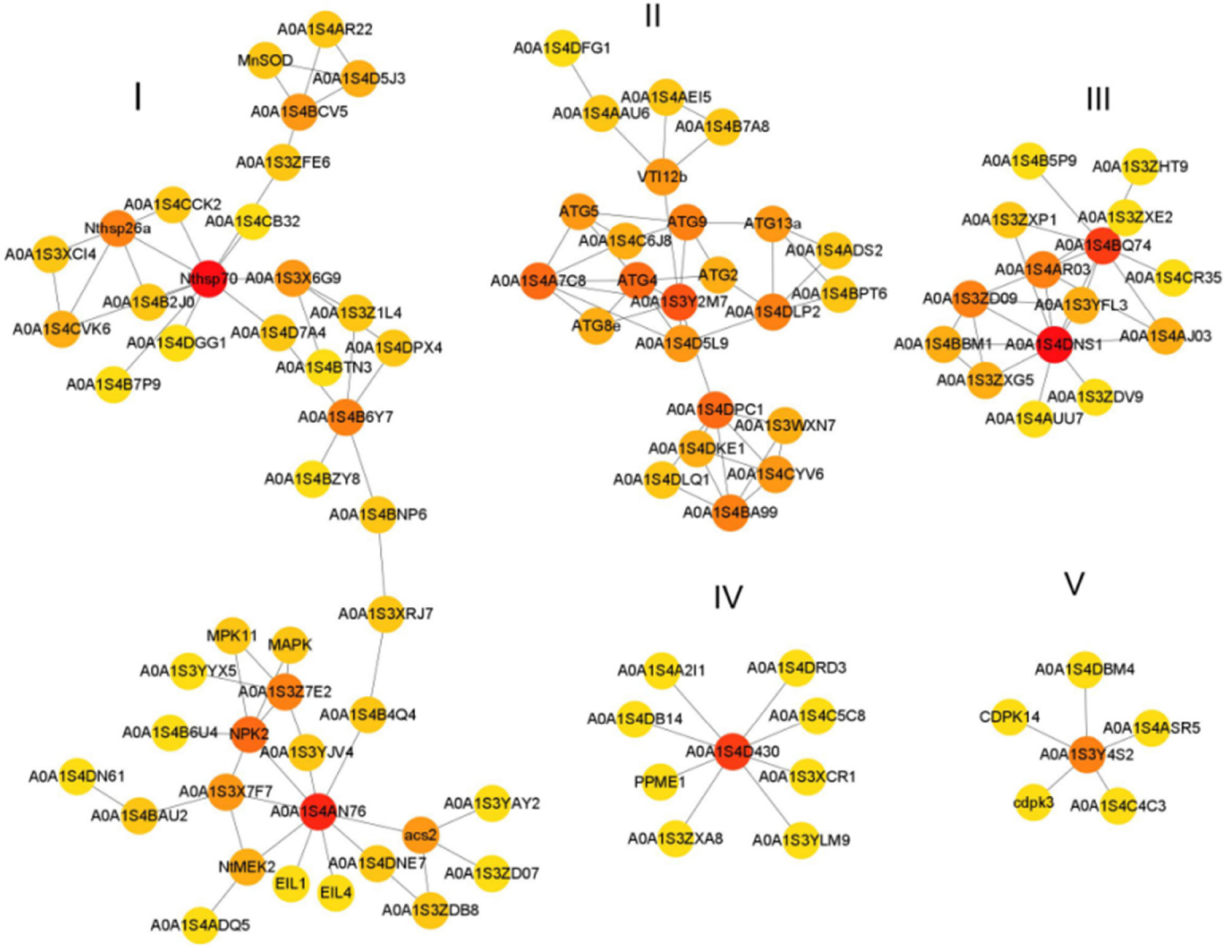
The network of protein–protein interaction displayed by common DEGs involved in replaceable bud PCD of chestnut cv. “Tima Zhenzhu.” Target proteins are identified by their gene names and represented by nodes. I-V represent five network modules.

To further investigate the regulatory pathways related to replaceable bud PCD of chestnut cv. “Tima Zhenzhu,” a protein–protein interaction network was completed for the DEGs associated with plant PCD, according to their tobacco homologs (Figure 7). The resultant network included 114 DEGs grouped into 5 distinct modules. The first network module was clustered into two groups: one consisting of heat-shock proteins (e.g., Nthsp70, A0A1S4B2J0, Nthsp26a, A0A1S4CVK6, A0A1S3XCI4, A0A1S4CCK2, and A0A1S4CB32), and the other consisting of DEGs involved in either the ethylene signaling pathway (e.g., EIL4, EIL1, A0A1S4DNE7, acs2, A0A1S3YAY2, A0A1S3ZD07, and A0A1S3ZDB8) or the MAPK signaling cascade (e.g., A0A1S4AN76, NtMEK2, A0A1S4ADQ5, NPK2, MPK11, A0A1S4B6U4). Notably, between the two groups, A0A1S4B6Y7 (metacaspase-9-like) showed strong interactions with A0A1S3Z1L4 (Ca^2+^-transporting ATPase), A0A1S4DPX4 (Ca^2+^-transporting ATPase), and A0A1S4BNP6 (chaperonin 60 subunit), suggesting that these genes may interact at the nexus of stress response and ethylene and MAPK signaling. The second network module consisted of autophagy-related proteins (A0A1S3Y2M7, ATG8e, A0A1S4A7C8, ATG4, A0A1S4D5L9, ATG5, A0A1S4C6J8, ATG9, ATG2, A0A1S4DLP2, ATG13a, and A0A1S4BPT6) and vacuolar protein sorting-associated proteins (A0A1S4DPC1, A0A1S4DKE1, A0A1S4CYV6, A0A1S4BA99, A0A1S3WXN7, and A0A1S4DLQ1). The third network module consisted of DNA repair proteins (e.g., A0A1S3ZD09, A0A1S3ZXG5, A0A1S4BBM1, and A0A1S3YFL3) and exonucleases (A0A1S4AJ03, A0A1S3ZDV9, and A0A1S4AUU7). In the fourth network module, A0A1S4D430, a polygalacturonase-like protein, showed strong interactions with eight pectinesterase proteins (PPME1, A0A1S4DRD3, A0A1S3XCR1, A0A1S4A2I1, A0A1S4C5C8, A0A1S3ZXA8, A0A1S4DB14, and A0A1S3YLM9). In the fifth network module, A0A1S3Y4S2, a respiratory burst oxidase homolog protein (RBOH), showed strong interactions with several Ca^2+^-dependent protein kinases (CDPKs) (e.g., A0A1S4C4C3, CDPK14, A0A1S4DBM4, cdpk3, and A0A1S4ASR5).

### 3.6 qRT-PCR validation of the transcriptomic data

**Figure 8:**
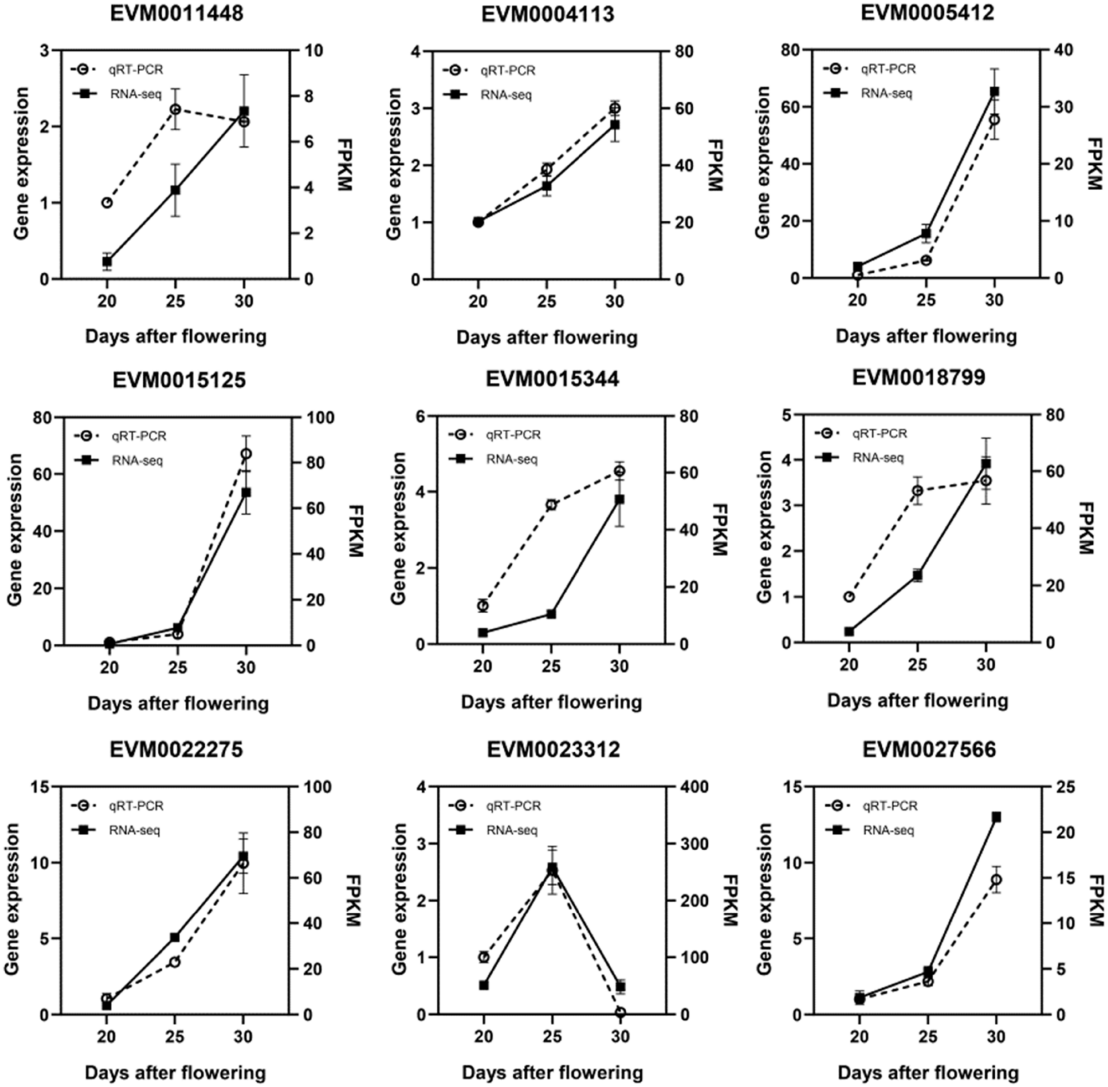
qRT-PCR validation of DEG expression levels obtained from RNA-seq.

Nine PCD-related DEGs were chosen for further qRT-PCR analysis to confirm their expression levels during replaceable bud PCD of chestnut cv. “Tima Zhenzhu.” Overall, the RNA-seq results were largely in agreement with the qRT-PCR results (Figure 8), suggesting that our transcriptomic analysis was reasonable and accurate.

## 4 Discussion

### 4.1 Role of the ethylene signaling in replaceable bud PCD of chestnut cv. “Tima Zhenzhu”

Phytohormone signal transduction is likely the prevailing mechanism responsible for the upstream regulation of PCD processes [7]. For example, ethylene is responsible for leaf senescence and root deterioration in maize [10,11]; auxin is responsible for developmentally regulated PCD in lace plants [20]; GA is related to aleuronal PCD [21]; and the combination of auxin and BR signal transduction may indirectly mediate PCD to regulate pericarp thickness in the sweet corn [22]. Here, KEGG analysis also indicated that the “plant hormone signal transduction” (ko04075) pathway was particularly enriched. Several key components of the ethylene signaling pathway were found to be up-regulated at S25, including ERS1 (EVM0016645) and EIN3 (EVM0013705, EVM0023854, and EVM0025637) (Figure 6). In particular, EIN3 exerts control over cellular senescence and death through its involvement in a trifurcate feed-forward pathway [23]. Within the EIN2–EIN3–NAC regulatory cascade controlling leaf senescence-associated PCD, EIN3 acts to transcriptionally activate NAC TFs (ORE1 and AtNAP), which themselves act to positively regulate leaf senescence [24]. Additionally, EIN3 functions as a direct repressor of miR164 [23], which acts to post-transcriptionally negatively regulate *ORE1*. In order to efficiently regulate leaf senescence, EIN3 appears to simultaneously regulate the expression of both *ORE1* and its particular negative regulator (miR164). By analogy, the upregulation of EIN3 at S25 likely promotes replaceable bud PCD. Taken together, these findings suggest that ethylene signaling is activated during preparation for PCD in replaceable buds of chestnut cv. “Tima Zhenzhu.”

In our previous study, we reported that the ethylene content was higher in replaceable buds at S25 than at S20 [25]. Our present study bolsters this observation, as we found that genes (encoding ACO and ACS) related to ethylene biosynthesis were consistently upregulated at S25 (Table S6). We identified additional DEGs involved in other hormone signaling pathways, suggesting that several phytohormones may interact during replaceable bud PCD. For example, both GA and ABA antagonistically regulate aleuronal PCD [5]. Additionally, leaf senescence is promoted by a combination of ethylene, SA, and ABA, while this process is delayed by a combination of auxin, cytokinin, and GA [26,27]. As shown in Table S5 and Figure 6, DEGs involved in ethylene signaling, such as MAPK (EVM0010215), ERS1 (EVM0016645), EIN3 (EVM0013705 and EVM0025637), and ERF1B (EVM0030131 and EVM0003409), were upregulated at S25 vs S20. Contrarily, DEGs involved in the auxin pathway, such as AUX28 (EVM0014325), SAUR-like auxin-responsive proteins (EVM0004337, EVM0007905, EVM0019733, and EVM0021087), and auxin-responsive proteins (e.g., EVM0003597, EVM0004089, EVM0005109, EVM0007851, EVM0017536, and EVM0017744), or the cytokinin pathway, such as AHP5 (EVM0015700) and two-component response regulator ARR family members (EVM0008810, EVM0017362, EVM0020552, and EVM0022760), were downregulated at this same time point. In our previous study, we reported that auxin and cytokinin content were significantly lower in replaceable buds at S25 than at S20 [25]. Taken together, it appears that replaceable bud PCD of chestnut cv. “Tima Zhenzhu” is primarily controlled by high ethylene and low auxin and cytokinin signaling, suggesting that ethylene may antagonistically interact with auxin and cytokinin in this process.

The MAPK signaling cascade acts to transduce extracellular signals into biochemical and physiological responses [28]. Each MAPK signaling cascade contains at least three essential members: MAPK, MAPKK, and MAPKKK [29]. Evidence shows that the MAPK signaling cascade may be essential for PCD in plants. For example, in mutant rice, several genes encoding MAPKs (e.g., *MPK3*, *MPK5*, and *MPK13*) are upregulated during leaf PCD [30]. MAPK appears to work to initiate self-incompatibility (SI)-induced PCD when poppy flowers are exposed to incompatible pollen [31]. In *Arabidopsis*, MAPK cascades regulate leaf senescence [32], mediate cell death, and induce ethylene production by regulating *ACS2*, *ACS6*, and *ACS8* gene expression [29]. Here, we identified 16 DEGs (e.g., EVM0012332, EVM0022934, EVM0004329, EVM0000127, and EVM0025130) associated with MAPK signaling, with most of these exhibiting upregulation during replaceable bud PCD (Table S6). Furthermore, a MAPK cascade involving A0A1S4ADQ5 (MAPKKK)–NtMEK2 (MAPKK)–A0A1S4AN76 (MAPK) was found in the protein–protein interaction network (Figure 7), and A0A1S4AN76 showed strong interactions with ethylene biosynthesis-related genes *A0A1S4DNE7* (1-aminocyclopropane-1-carboxylate synthase), *A0A1S3ZDB8* (S-adenosylmethionine synthase), *A0A1S3YAY2* (1-aminocyclopropane-1-carboxylate oxidase-like), *A0A1S3ZD07* (1-aminocyclopropane-1-carboxylate oxidase), and *acs2* (1-aminocyclopropane-1-carboxylate synthase-like). Taken together, we speculate that the MAPK cascade is involved in ethylene biosynthesis via differential regulation of related genes during replaceable bud PCD of chestnut cv. “Tima zhenzhu.”

### 4.2 PCD initiation in replaceable buds of chestnut cv. “Tima Zhenzhu”

Several TFs may act as bridges linking phytohormone signaling with PCD regulation [33]. As an example, ORESARA1 (ANAC092, a NAC TF) regulates leaf senescence both downstream of ethylene signaling and upstream of senescence-inducing genes, including NAC TFs such as BIFUNCTIONAL NUCLEASE 1 (BFN1) [34]. In this study, we discovered a large number of DEGs encoding TF homologs. These results suggest that several TFs may act to ensure the initiation of bud PCD of chestnut cv. “Tima Zhenzhu.”

Cytochrome c plays a part in the PCD signaling network, with the upregulation of both cytochrome c oxidase and cytochrome c being an early event during the process of PCD [35]. The process of PCD in several plants is accompanied by the dispensation of mitochondrial cytochrome c, including tapetal PCD in sunflowers and terminally differentiated suspensor PCD in runner bean cotyledons [36,37]. Here, we found that the expression levels of genes encoding cytochrome c (EVM0024076) and cytochrome c oxidases (EVM0029309, EVM0032493, EVM0012209, EVM0004365, EVM0022679, and EVM0012699) were notably upregulated at S25 compared to S20 (Table S6). Therefore, we suggest that cytochrome c and cytochrome c oxidases may modulate replaceable bud PCD initiation in chestnut cv. “Tima Zhenzhu.”

Substantial evidence indicates that Ca^2+^, a universal second messenger, is crucial for PCD regulation in plants. Cytoplasmic Ca^2+^-influx from vacuoles and endoplasmic reticulum is an early event during PCD [38]. Specifically, Ca^2+^-permeable channels, Ca^2+^ sensors including calcineurin B-like proteins (CBLs), calmodulins (CAMs), and CDPKs are necessary for Ca^2+^ signal transduction and PCD [39]. The activities of many enzymes and other proteins associated with various PCDs are under the control of Ca^2+^/CAM or Ca^2+^/CBL complex such as the Ca^2+^-dependent endonucleases and hydrolytic enzymes [40]. CDPK3 is a positive regulator of LCB (sphingoid long-chain bases)-mediated PCD in *Arabidopsis* [41]. In the present research, a total of 36 DEGs were found to be associated with Ca^2+^ signaling, consisting of 4 Ca^2+^ channels, 9 CDPKs, 9 calcineurin B-like proteins, and 14 calmodulins. Most of these DEGs exhibited similar expression patterns with significantly higher levels at S25 compared to S20 (Table S6), suggesting that cascade events are likely to contribute to the calcium signaling. Moreover, in our previous study, increased calcium levels were observed in the replaceable bud cells undergoing PCD in chestnut cv. “Tima Zhenzhu” [42]. Therefore, it is reasonable to infer that the calcium-dependent signaling cascade should be related to modulating the replaceable bud PCD initiation in chestnut cv. “Tima Zhenzhu.” Furthermore, in tomatoes, Ca^2+^-CBL10/CIPK6 complex promotes the accumulation of ROS by activating the respiratory burst homolog RBOH and hence regulates the process of PCD [43]. Similar results were found in the present research: in the constructed protein–protein interaction network (Figure 8), A0A1S3Y4S2, an RBOH, showed strong interactions with five CDPKs, and both RBOH and CDPKs exhibited upregulated expression with significantly higher levels at S25 and S30 compared to S20, these results suggest that the CDPKs may promote the accumulation of ROS by activating RBOH, which may also be involved in the downstream cascade of Ca^2+^ regulatory pathway in replaceable bud PCD of chestnut cv. “Tima Zhenzhu.”

### 4.3 PCD execution in replaceable buds of chestnut cv. “Tima Zhenzhu”

The reception of specific signal triggers initiates PCD execution and cellular corpse clearance, activating and releasing myriad hydrolytic enzymes, including various nucleases and proteases [33]. Our previous study found no evidence of DNA degradation at S20, with some DNA degradation at S25 and heavy DNA degradation at S30 [1]. Here, we found two DEG encoding nucleases, including endonuclease V isoform X1 (EVM0021509) and endonuclease 1-like isoform X2 (EVM0022275), which were significantly upregulated at S25 and S30 compared to S20 (Figure 8; Table S6), may promote the DNA degradation of bud PCD in chestnut cv. “Tima Zhenzhu.” We also found a variety of DEGs (upregulated at S25 and S30 compared to S20) encoding proteases, including cysteine proteinases RD21A-like, metacaspase-9-like, aspartic proteinases, vacuolar-processing enzyme-like, senescence-associated proteins, endoglucanases, pectinesterase, xyloglucan endotransglucosylase/hydrolases, xyloglucan galactosyltransferase, and exosome complex component RRP45A-like. Previous studies on homologs of our identified cysteine proteinases RD21A-like, metacaspase-9-like, aspartic proteinases, vacuolar-processing enzyme-like, and senescence-associated proteins suggest that these enzymes are crucial for effective PCD by degrading many essential cellular targets [14,19,33,44]. Endoglucanases, pectinesterases, xyloglucan endotransglucosylase/hydrolases, and xyloglucan galactosyltransferases may act to recycle carbohydrates as cells undergo PCD. Finally, homologs of complex exosome component RRP45A-like likely participate in DNA degradation [13].

Autophagy is a metabolic mechanism whereby cytoplasmic substances and organelles are degraded by lysosomes or vacuoles and is a requirement for efficient PCD in many plant systems [45]. Autophagic cell death is indicated by several distinct morphological features in plants, such as an increase in vacuolar and cellular size, movement of organelles into the vacuole for destruction, and subsequent cell death resulting from vacuolar lysis [46,47]. Autophagy plays both pro-survival and pro-death roles in plant PCD [47]. The core autophagy mechanism is organized by an evolutionarily conserved ATG (AuTophaGy-related) gene population [46]. In our previous study, we found that during replaceable bud PCD of chestnut cv. “Tima Zhenzhu,” small vacuoles in the cytoplasm fused to form a large vacuole, resulting in the eventual degradation of other CCs [1]. Here, in accordance with this observation, we found that “regulation of autophagy” pathway genes were upregulated at S25 and S30 compared to S20, including autophagy-related proteins 8f, 16, 8i-like, 8C-like, 3 and 13a (EVM0027149, EVM0009961, EVM0003991, EVM0004768, EVM0015502, EVM0002553, EVM0027459, and EVM0017527), cysteine protease ATG4-like (EVM0001368), ubiquitin-like modifier-activating enzyme ATG7 (EVM0017393), ubiquitin-like protein ATG12 (EVM0018088), and CBL-interacting serine/threonine–protein kinase 1-like and 6-like (EVM0001436 and EVM0010887) (Table S9). Additionally, the highly selective autophagy of soluble proteins is mediated by the ESCRT (endosomal sorting complex required for transport) complex, which requires vacuolar protein sorting (VPS) [48]. We found six DEGs (five up- and one downregulated at S25 and S30 compared to S20) encoding VPS-associated proteins (A0A1S4DPC1, A0A1S4DKE1, A0A1S4CYV6, A0A1S4BA99, A0A1S3WXN7, and A0A1S4DLQ1) which interacted with each other and with the network of autophagy-related proteins (A0A1S3Y2M7, ATG8e, A0A1S4A7C8, ATG4, A0A1S4D5L9, ATG5, A0A1S4C6J8, ATG9, ATG2, A0A1S4DLP2, ATG13a, and A0A1S4BPT6) (Figure 7; Table S8). Autophagic processes are necessary for the timely progression of replaceable bud PCD of chestnut cv. ‘Tima Zhenzhu,’ and that replaceable bud PCD may depend on VPS-associated proteins.

## 5 Conclusions

**Figure 9:**
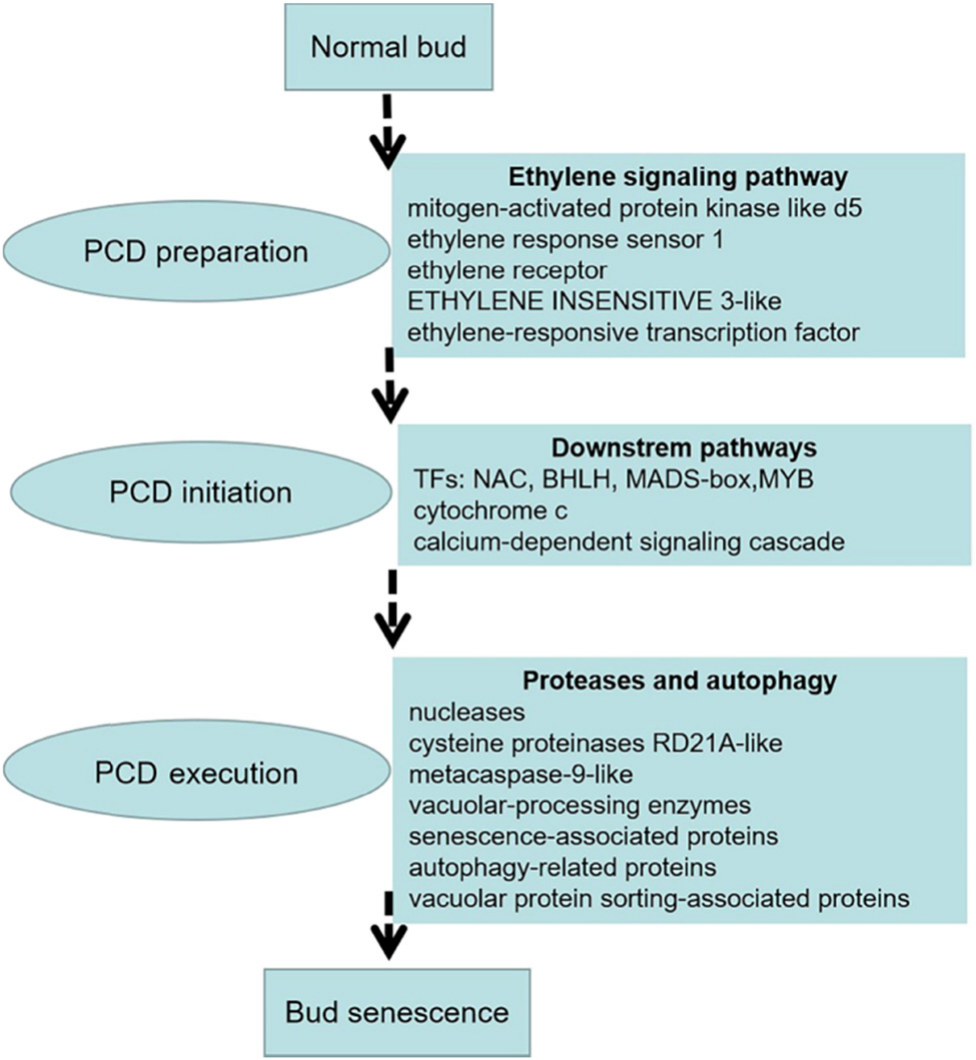
Tentative model of PCD during replaceable bud senescence in chestnut cv. “Tima Zhenzhu.”

We identified the DEGs and signaling pathways responsible for regulating replaceable bud PCD in chestnut cv. “Tima Zhenzhu” through transcriptomic profiling. Based on our cumulative results, we offer a hypothetical model of replaceable bud PCD consisting of three overlapping processes (Figure 9). First, ethylene signaling is activated during preparation for PCD in order to regulate the activity of downstream targets. Next, during PCD initiation, the up-regulation of several TFs (including MYB, MADS-box, bHLH, and NAC TFs) induces an increase in cytochrome c expression and the cytosolic Ca^2+^ content, activating the Ca^2+^-dependent signaling cascade. Finally, during PCD execution, the process of autophagy and several proteases (i.e., cysteine proteinases RD21A-like, metacaspase-9-like, vacuolar-processing enzyme-like, and senescence-associated proteins) work synergistically to clear the cell of CCs. When this process is complete, the replaceable bud senesces and dies. This hypothetical model will bolster our understanding of the molecular mechanism of bud PCD. In the later stage, we need to do further research on gene function and gene interaction through transgenic systems, yeast two-hybrid systems, and other technical methods.

## Supplementary Material

Supplementary Table
